# The Natural History of Uterine Venous Plexus Thrombosis

**DOI:** 10.3390/diagnostics11081338

**Published:** 2021-07-26

**Authors:** Tejal N. Amin, Hannah Cohen, Michael Wong, Sara-Louise Pointer, Naaila Aslam, Davor Jurkovic

**Affiliations:** 1 Institute for Women’s Health, University College London Hospitals NHS Foundation Trust, London WC1H 8NJ, UK; michael.wong3@nhs.net (M.W.); sara-louise.pointer@nhs.net (S.-L.P.); naaila.aslam@nhs.net (N.A.); davor.jurkovic@nhs.net (D.J.); 2 Department of Haematology, University College London Hospitals NHS Foundations Trust, London WC1H 8NJ, UK; hannah.cohen@ucl.ac.uk; 3 Haemostasis Research Unit, Department of Haematology, University College London, London WC1H 8NJ, UK

**Keywords:** venous thromboembolism, deep vein thrombosis, ultrasound, uterine vein plexus thrombosis, management, resolution

## Abstract

The management of incidental or unusual site venous thrombosis (VT) is challenging and is often extrapolated from studies on symptomatic deep venous thrombosis (DVT). There is a tendency to treat with anticoagulation, due to the theoretical risk of propagation and embolism; however, this is not without risk. Furthermore, there is little guidance on how to monitor incidental VT. The aim of this study was to describe the natural history of incidental uterine venous plexus thrombosis (UVPT) and provide a structured approach to its overall management. A prospective study was conducted in a university teaching hospital over a 16-month period. Women diagnosed with UVPT on transvaginal ultrasound (TVS) were followed up over a six-month period and managed based on an individualised risk assessments, in conjunction with haematologists. Fifty women were diagnosed with UVPT during the study period, of which 38 were managed expectantly. The resolution was documented in 70% of women. There were no cases of symptomatic DVT or pulmonary embolisms in either the expectant or treatment groups. Our study has shown that in a high proportion of women, incidental UVPT could be managed successfully without the need for anticoagulation. The overall management of UVPT should be based on individualised clinical risk assessments.

## 1. Introduction

Management of incidental, asymptomatic venous thrombosis (VT), or unusual site VT, remains challenging and there are no substantive data to guide whether or not anticoagulation is required. Studies have shown that the risk of propagation and embolism is low with incidental calf deep venous thrombosis (DVT) [1], however management is often extrapolated from studies on symptomatic DVT. Our previous study has shown that the prevalence of uterine venous plexus thrombosis (UVPT) is approximately 3% in women attending gynaecological clinics [2]. Once diagnosed, UVPT can present clinicians with a management dilemma. Such thrombosis can theoretically embolise into the pulmonary circulation, remain insitu, and evolve into pelvic phleboliths (or act as a precursor for valvular incompetence, leading to pelvic varicosities). Given the paucity of data on UVPT, there has been a tendency to treat with anticoagulation [3,4], in women considered to be at a potentially higher risk of venous thromboembolism (VTE), for example women who became pregnant during follow up or are due to have surgery or long-haul travel [4]. However, anticoagulation is not without risk, and the treatment of asymptomatic DVT can result in a higher risk for major bleeding, without a significant effect on the incidence of recurrent symptomatic VTE [5]. The aim of this study was to describe the natural history of asymptomatic UVPT and provide a structured approach to its overall management.

## 2. Methods

This was a single centre, prospective cross-sectional study of women attending our gynaecology clinic from August 2015 to December 2016. All women who attend the gynaecology clinic undergo a transvaginal scan (TVS), as part of their routine clinical assessment. Women were recruited into the study if they were diagnosed with UVPT during their routine TVS examination. We excluded women who were <18 years old, pregnant at the time of the first ultrasound examination, declined/could not undergo TVS, and/or had previously undergone a hysterectomy. The study was approved by the West Midlands-Solihull national ethics committee (14/WM/1266) and entered onto the ISRCTN registry (No.15750232). Written consent was obtained from all women prior to the examination. Women diagnosed with UVPT were also provided written patient information leaflets about the condition.

**Figure 1:**
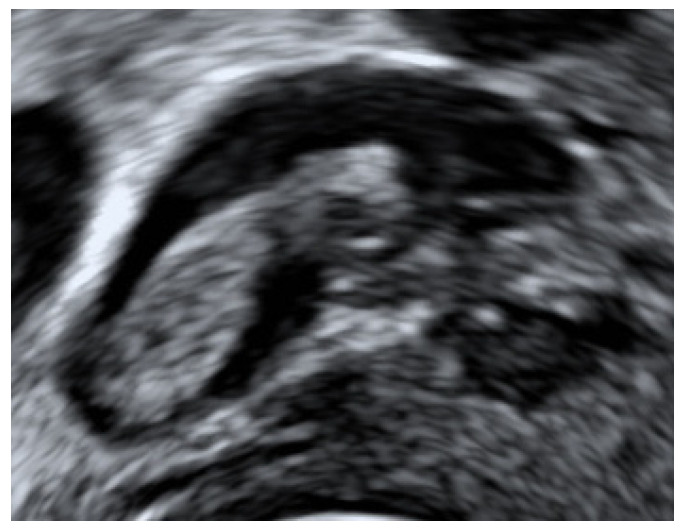
Transvaginal B-mode image of uterine vein thrombus.

All women underwent TVS, which was performed using a standardised technique, as previously described [6]. In brief, once the examination of pelvic organs had been completed, the uterine venous trunks were located in the broad ligament in the vicinity of the internal cervical os. We used colour Doppler to differentiate between uterine veins and other tubular structures within the broad ligament, such as arteries and ureters. The diagnosis of a thrombus was based on pre-defined, established criterion, including the presence of a solid, hyperechoic intraluminal structure causing partial venous blood flow obstruction [4] (Figure 1). The largest thrombus dimensions were measured in three orthogonal planes, and the mean diameter was taken as the final value [6]. The velocity and type of flow was also recorded using pulsed-wave (PW) Doppler, with the angle of insonation set to <60 degrees. All diagnoses of UVPT were verified by Level III gynaecological ultrasound operators (D.J. and N.A.). The images were also reviewed independently by a third level III operator with experience in the ultrasound diagnosis of deep vein thrombosis (S-L.P.).

**Figure 2:**
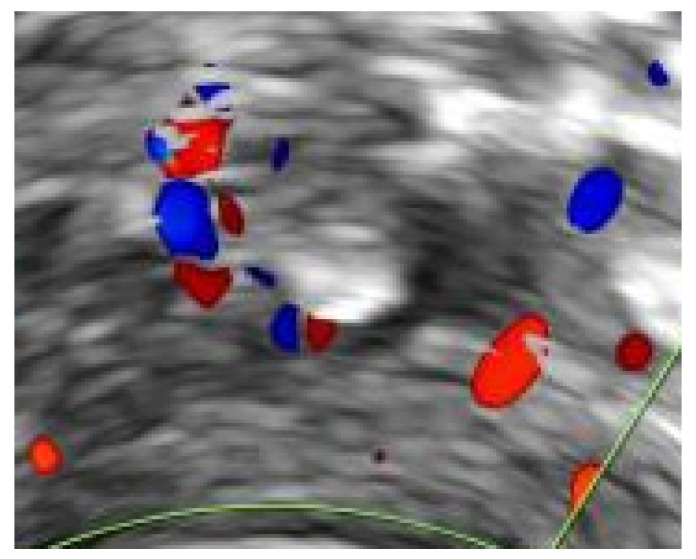
Transvaginal image of uterine vein phlebolith with colour Doppler.

**Figure 3:**
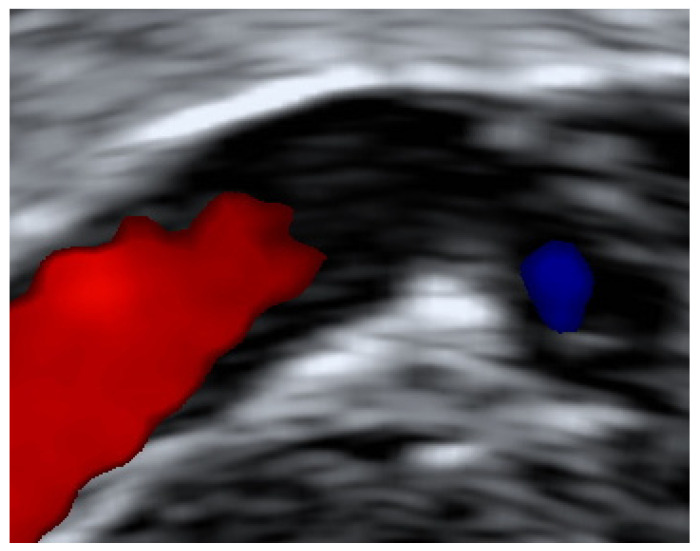
Colour Doppler transvaginal image of fibrosis within uterine vein.

All women diagnosed with UVPT were invited to attend for a follow up scan four weeks later. In women with persistent thrombosis, additional follow up scans were arranged at three and six months. The resolution of UVPT was confirmed when there was no evidence of a residual solid thrombus. Women who had evidence of fibrosis in the vessel wall at the site of the UVPT, or the presence of phleboliths, were included in the resolution category (Figure 2 and Figure 3).

In addition to the TVS examination, all women diagnosed with UVPT had baseline thrombophilia screens and lower limb venous duplex scans to exclude concomitant lower limb DVT. They were referred to a consultant haematologist (H.C.) specialising in thrombosis and haemostasis for assessment, as well as clinical advice on management and risk-reduction measures against thrombosis as per routine clinical practice, based on the study protocol, which has been described previously [2]. Women considered to be at a potentially higher risk for VTE, as defined above, were advised to have thromboprophylactic low-molecular weight heparin (LMWH). Those with abnormal thrombophilia results were assessed on an individualised basis and offered prophylactic or treatment dose LMWH, depending on the type of abnormality and its associated risk. Those who had normal thrombophilia profiles and lower limb venous duplex Doppler scans were offered expectant management and followed up as per the protocol, with the option to choose treatment at any point.

### Statistical Analysis

The resolution analysis was performed at the person level. For women with multiple thrombi, resolution was defined as having occurred at the time-point where all the clots were resolved. Patients who conceived during the study were censored at the last time point. The Kaplan-Meier survival analysis was used to examine the resolution over the six-month surveillance period and was presented as the number of women still affected by persistent clots at each time point, as well as the estimate of the unresolved rate with corresponding confidence intervals (CI). Cox regression analysis was used to examine the associations with time to clot resolution. The exception was for variables where there was no resolution in one (or more) categories, where it is not possible to perform Cox regression. The logrank test was used in such instances. The separate univariable association between each parameter and clot resolution time was examined. The size of the association between each factor and outcome is presented as a hazard ratio, along with corresponding CI and *p*-values. For the categorical variables, this reflects the hazard or chance of resolution in each category, relative to the hazard in a baseline category. For the continuous variables, the hazard ratios give the relative change in the chance of resolution for one-unit increase in the variable.

## 3. Results

### 3.1. Patients

During the study period, 50 women were diagnosed with UVPT. Patient demographics and risks factors are presented in the following table (Table 1). Of the 50 women diagnosed with UVPT, 19 (38%) had raised D-dimer levels and 14 (28%) had abnormal initial thrombophilia screens: one mild protein C deficiency, one mild protein S deficiency, six were homozygous, and nine heterozygous for the C655T methylene tetrahydrofolate reductase (MTHFR) polymorphism. None had antithrombin deficiency, factor V Leiden (FVL), or the prothrombin G20210A gene mutation (PGM). One patient was positive for lupus anticoagulant, four women had moderate titre IgM anti-cardiolipin antibodies (aCL), and two had moderate titre IgM anti-beta-2 glycoprotein 1 antibodies (aß2GPI). Mildly elevated total homocysteine was observed in 14 women (28%).

**Figure 4:**
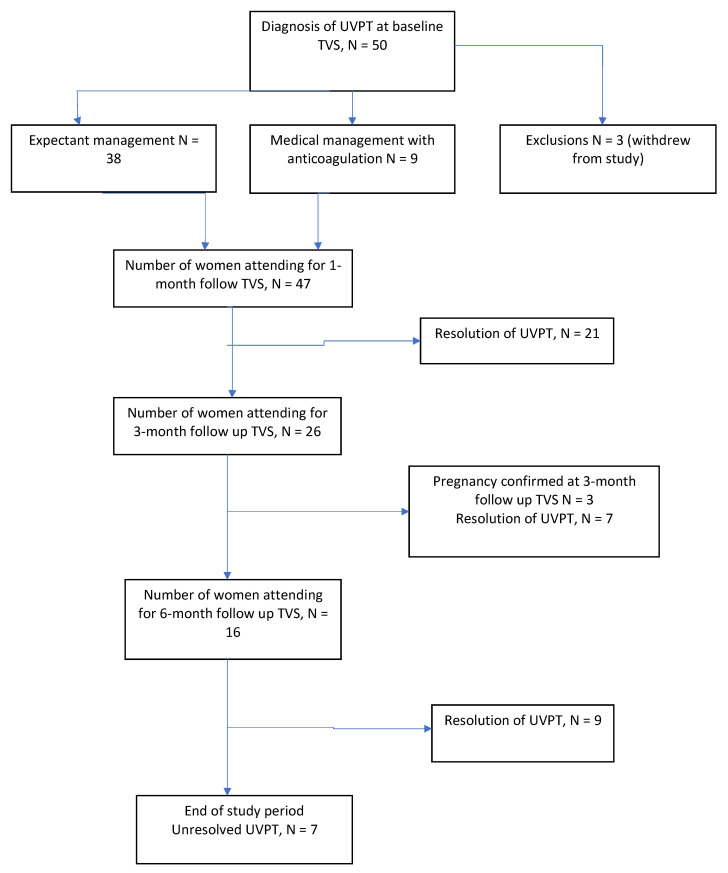
Flow chart of study participants.

Thrombosis was unilateral on the left side in 24 women (48%), unilateral on the right side in 20 women (40%), and bilateral in 6 women (12%). The mean diameter of the thrombi at diagnosis was 8.87 mm (range 1.7 mm–15.5 mm). Four women (8%) had uterine veins that were >95th centile for their age/parity, which could suggest underlying pelvic varicosities. Three women withdrew from the study before the one-month assessment and were excluded from the resolution analysis. They were all given clinical advice on the management and risk-reduction measures against thrombosis in the haematology clinic and were managed outside of the study protocol. The remaining 47 women were included in the analysis. Three women conceived during the study period and were censored at the time of the last follow up. These women were commenced on prophylactic LMWH and managed in the joint obstetric/haematology clinics (Figure 4).

### 3.2. Management

Expectant management was used in 38 women (81%), while the remaining nine women (19%) received anticoagulation based on the findings at the initial examination. Four women received a treatment dose of LMWH for six weeks, followed by 3 months of apixiban (2.5 mg twice daily). The treatment dose anticoagulation was given for the following reasons: a positive finding of a soleal vein thrombus in the contralateral leg to the UVPT in one woman, known antiphospholipid syndrome (APS) and recent long-haul travel in another woman, multiple thrombi and recent surgery in the third woman, and a diagnosis of breast cancer four months prior to the examination and tamoxifen treatment in the fourth patient. Five women received a prophylactic dose of LMWH for up to eight weeks, based on individualised risk assessments, which included a positive high-risk thrombophilia screen, recent/upcoming surgery, or long-haul travel.

### 3.3. Resolution of UVPT

**Figure 5:**
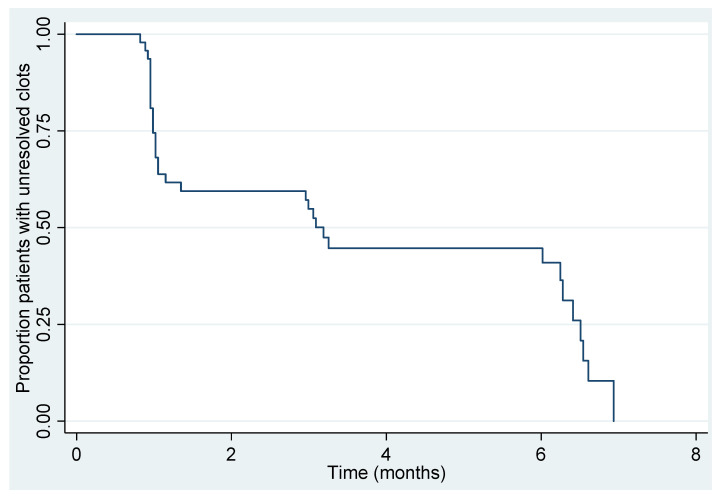
Kaplan-Meier plot of time to resolution for all women (*n* =47).

**Figure 6:**
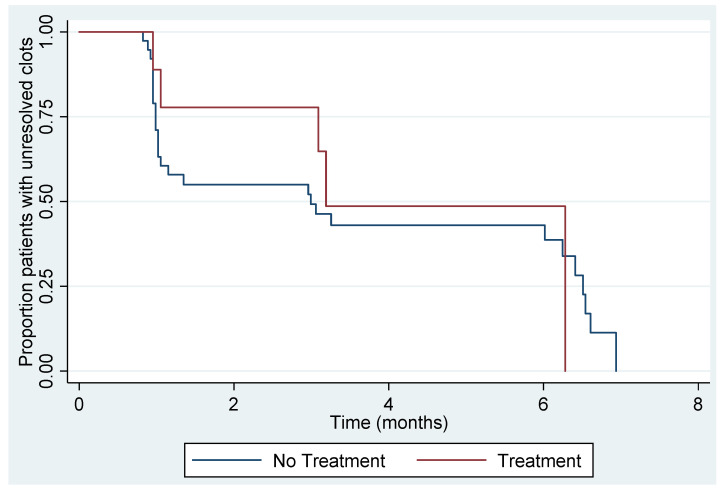
Kaplan-Meier plot of time to resolution between women who were managed expectantly (*n* = 38) and with anticoagulation (*n* = 9).

Resolution was calculated on the sample of 47 women who were included in the final analysis. At the end of the six-month follow up period, 33 out of 47 (70%, 95% CI 55–83) women had all of the thrombi resolved. Out of the 38 women who were managed expectantly, 28 (74%, 95% CI 57–87) women had resolution of their thrombi. None of the women who were managed expectantly demonstrated thrombus extension on the follow up surveillance scans. Within the resolution group, five women had received anticoagulation: three with prophylactic doses of LMWH and two with treatment dose of LMWH. Four women who had received prophylactic, or treatment dose anticoagulation, demonstrated persistent thrombi at the six-month ultrasound scan, which were hyperechoic and chronic in appearance. Figure 5 illustrates the proportion of unresolved thrombi at one, three, and six months overall, with a numerical summary in Table 2. The difference in resolution between the expectant management and treatment groups is shown in Figure 6. The results suggest that at one-month, over one-half of women had unresolved thrombi, which then reduced to below 50% after the three-month checkpoint. We did not identify any factors that were strongly associated with resolution (Table 3). Due to the small numbers of UVPT patients, no multivariable analysis was performed.

## 4. Discussion

In this prospective cross-sectional study of the natural history of asymptomatic UVPT, we found that by the end of the six-month follow up period, 70% of the 47 women studied had resolution of their thrombi. There was no difference in the overall resolution rates between women who received anticoagulation and those who had expectant management without anticoagulation. We found that there were several risk factors associated with the development of UVPT, including multiparity, pre-menopausal status, a recent surgery within four months, the presence of varicose veins of the legs, and a family history of VTE in a first-degree relative [2]. However, we did not find any significant factors that were associated with resolution. There were no cases of symptomatic pulmonary embolism in the expectant or treatment groups.

The resolution and recanalization of venous thrombosis generally begins within one week of an acute episode [7], however the rates can vary for several reasons, such as the type of imaging modality used and frequency of surveillance. The overall management with and without anticoagulation can also influence the resolution. In a study by Masuda et al. [1], complete lysis of calf vein thrombosis was demonstrated in 88% of patients at three months, of which approximately 48% had not received anticoagulation. O’Shaughnessy et al. [8] found that there was complete resolution of venous thrombosis in 60% of their patient population in one year. Another study reported the complete resolution of venous thrombosis occurring anytime between five to 170 days [9]. We found that by the six-month follow up period, UVPT had resolved in over 70% of women. Other studies have reported that if thrombus resolution or vein recanalization is to occur, it should be evident in the first six months [10], which supports that recanalization is a slow process [11]. It is, therefore, likely that in our study, some of the thrombi diagnosed may have been older, chronic, and in the organization and recanalization phase.

Previous studies have reported that resolution can be dependent on the thrombus load, with faster recanalization and resolution rates in legs where one venous segment is affected, compared to thrombi involving multiple segments [12,13]. We found in our study that there was no overall difference in resolution between women with single or multiple UVPT or the initial size of the thrombus, but this could be due to the small numbers in the anticoagulation group. Of note, there were a relatively small number of resolutions, and, thus, relatively low power to detect significant associations.

Anticoagulation is the mainstay treatment for symptomatic VTE, as it prevents further thrombus deposition and extension, allows stabilization and/or lysis of the established thrombus, and reduces the risk of interval thrombosis [11]. In our study, we found that there was no significant effect of anticoagulation on the overall clot resolution (*p* = 0.64). The initial clot diameter was not associated with the time to resolution. There are limited data investigating whether the initial thrombus size has an effect on the overall resolution. Sartori et al. [14] found that the recanalization rate was not correlated to calf vein thrombus diameter at enrolment (*p* = 0.93), and there was no difference in recanalization rates between clots that were <5 mm and ≥5 mm.

In our study, we also found that the presence of a higher or lower-risk thrombophilia did not influence resolution, which has been shown to be identical between patients with and without thrombophilia [15]. D-dimer level at diagnosis was also found to not have an effect on time to resolution. Although D-dimer testing is incorporated into DVT diagnostic algorithm [16], some studies have demonstrated that D-dimer levels can be normal in the presence of a DVT [17,18].

This is the first study to examine to report the natural course of UVPT in a non-pregnant population who have been managed expectantly. Very few studies have examined the course of pelvic vein thrombosis [3,4,19] and as with lower limb DVT, there may be a concern regarding propagation and embolism. In our study, we did not have any cases of symptomatic pulmonary embolism (PE), and only one woman had evidence of DVT in the contralateral leg. Labroulous et al. [20] studied symptomatic ovarian vein thrombosis (OVT) in 23 women over a 9-year period, in which the majority of their patients were postpartum and all were anticoagulated. The propagation rate of OVT into the IVC was 13%, and two women had evidence of PE. In addition, they found that three women had evidence of valvular incompetence. Similar studies have also been conducted for isolated calf DVT [1,17,21]. Masuda et al. [1] demonstrated that there were no cases of symptomatic PE in their non-treatment group, which is similar to our study findings.

The main strength of this study is that it is novel and the prospective study design permitted the evaluation of the natural history of UVPT. This current study, and our previous work on the prevalence of UVPT [2], can provide clinicians with a preliminary management pathway to follow. We have provided information that can be used to counsel patients diagnosed with UVPT on the clinical significance and likelihood of resolution. Although the absolute numbers of UVPT are small, this is still the largest and only study to prospectively follow up women with UVPT over a specified period of time. A limitation of this study was that we did not continue to scan women with persistent thrombi after the six-month surveillance period (or once the thrombus had resolved). Therefore, we were unable to ascertain whether these persistent thrombi may have calcified into pelvic phleboliths, which are commonly attached to the uterine veins and can be visualised on plain radiographs [22,23], or whether there was any evidence of recurrent UVPT, persistently dilated venous diameters, or venous incompetence that could precipitate pelvic varicosities. In our study, we had four women who had uterine veins >95th centile for their age and parity, which could be considered dilated [6]. However, we have not been able to determine whether the dilated veins act as a pre-cursor for the development of UVPT or are a consequence of UVPT. Further studies evaluating the uterine venous circulation are needed. The diagnosis of thrombosis can be anxiety provoking, which can be seen as a potential limitation. However, we were able to overcome this by providing information leaflets, a point of contact, and counselling from both the gynaecology and haematology teams.

Our study has shown that in a high proportion of women, incidental UVPT could be managed expectantly without the need for anticoagulation, but the overall management of UVPT should be based on the individualised clinical risk assessment. Adopting a multi-disciplinary approach between haematology and gynaecology provides a supportive structure for both clinicians and patients in managing this condition, which has a reported prevalence of 3% [2]. Given that post-thrombotic syndrome (PTS) can develop within two years following acute DVT [24], further prospective studies on the long-term follow up of women with UVPT are warranted, as well as establishing whether this could be a source of pelvic pain and/or pulmonary embolism.

## Figures and Tables

**Table 1:** Patient characteristics, medical, and ultrasound risk factors in the UVPT group.

Demographic Risk Factors	UVPT (*n* = 50)
Age (years)	44 (21–81 years)
Parity	
0	9 (18%)
1	11 (22%)
2	11 (22%)
3+	19 (38%)
BMI category	
Normal weight (BMI 18.5–24.9)	26 (52%)
Overweight (BMI 25–29.9)	10 (20%)
Obese (BMI > 30)	14 (28%)
Smoking	
Never	31 (62%)
Current/Former	8 (16%)
Ethnicity	
White	32 (64%)
Asian	3 (6%)
Black	11 (22%)
Middle Eastern	2 (4%)
Mixed/Other	2 (4%)
Menopausal status	
Pre-menopausal	43 (86%)
Post-menopausal	7 (14%)
Medical risk factors	
Current Hormone use ^(#)^	10 (20%)
Recent surgery (within four months)	7 (14%)
Recent immobilisation/travel (within six weeks)	3 (6%)
Personal history of thrombophilia ^(†)^	2 (4%)
Personal history of VTE (*)	1 (2%)
Family history of DVT/PE (**)	2 (4%)
Varicose veins of the legs	13 (26%)
Pelvic abnormalities on ultrasound as risk factors	
Fibroids	12 (24%)
Adenomyosis	20 (40%)
Adnexal/ovarian cyst (all ipsilateral)	3 (6%)
History or diagnosis of endometriosis	3 (6%)
Uterine vein diameter > 95th centile for age/parity	4 (8%)

^#^ including combined oral contraceptive pill, exogenous progestogens, hormone replacement therapy. ^†^ protein S and lupus anticoagulant (confirmed on repeat testing). * distal lower leg DVT. ** First-degree relatives only. Abbreviations: BMI = body mass index; DVT = deep venous thrombosis; PE = pulmonary embolism; VTE = venous thromboembolism.

**Table 2:** Summary of unresolved rates over time.

Timepoint	Women at Risk (N)	Kaplan-Meier Unresolved Estimate (95% CI)
0 months	47	-
1 months	26	59% (44%, 72%)
3 months	16	45% (30%, 59%)
6 months	7	15% (4% to 33%)

**Table 3:** Factors associated with clot resolution.

Variable	Category	Resolved *n*/N	Hazard Ratio (95% CI)	*p*-Value
Treatment with anticoagulation	No	28/38	1	0.64
	Yes	5/9	0.79 (0.30, 2.09)	
Initial diameter of thrombus (mm) ^(+)^	-	-	0.95 (0.84, 1.06)	0.36
Abnormal	No	19/23	1	0.69
thrombophilia (any)	Yes	14/24	0.86 (0.41, 1.79)	
D-dimer level (diagnosis) ^(^***^)^	-	-	1.00 (0.92, 1.08)	0.92
Age ^(^**^)^	-	-	1.01 (0.70, 1.45)	0.96
Parity	0	5/8	1	0.80
	1–2	16/20	1.15 (0.42, 3.20)	
	3+	12/19	0.89 (0.31, 2.56)	
BMI ^(^*^)^	-	-	0.85 (0.64, 1.14)	0.28
Family history DVT	No	32/45	1	0.76
	Yes	1/2	0.73 (0.10, 5.45)	
Menopausal status	Pre	29/40	1	0.66
	Post	4/7	1.27 (0.44, 3.70)	
Number of clots	Single	28/38	1	0.42
	Multiple	4/5	0.67 (0.26, 1.76)	
Vessel diameter ^(#)^	-		0.87 (0.72, 1.06)	0.17
Flow velocity ^(#)^	<5 cm/s	24/35	1	0.94
	≥5 cm/s	9/12	0.97 (0.43, 2.16)	

^(^*^)^ Hazard ratios given for a 5-unit increase in variable. ^(^**^)^ Hazard ratios given for a 10-unit increase in variable. ^(^***^)^ Hazard ratios given for a 100-unit increase in variable. ^(#)^ Value from affected side used in the analysis. ^(+)^ Size of largest clot if more than one. Abbreviations: BMI = body mass index; DVT = deep venous thrombosis.

## Data Availability

The data presented in this study are available on request from the corresponding author. The data are not publicly available due to the fact that it is contained within personal records of patients.
